# OT Bridge prosthetic system with different number of screws in all on six maxillary prosthesis: finite element analysis

**DOI:** 10.1186/s12903-025-06199-5

**Published:** 2025-05-28

**Authors:** Sara Ibrahim Soliman Mohamed, Shymaa Ibrahim Alattar, Abdulrhman Atef Bakry, Shaimaa Lofty Mohamed Ouda

**Affiliations:** 1 Oral and Maxillofacial Prosthodontics, Faculty of Dentistry, Ain-Shams University, Cairo, Egypt; 2 Tanta University, Tanta, Egypt; 3 Department of Oral Medicine and Removable Prosthodontics, Mansoura University, Mansoura, Egypt; 4 Faculty of Engineering, The British University in Egypt, Cairo, Egypt; 5 Department of Mechanical Engineering, The British University in Egypt, Cairo, Egypt; 6 114 Degla Gardens 6th of October, Giza, Egypt

**Keywords:** Screw-retained prosthesis, Screws number, All-on-six, Maxillary implant rehabilitation, Finite element analysis, OT Bridge system, Stress distribution

## Abstract

**Background:**

The OT-Bridge is reported as a simple versatile rehabilitation technique that guarantees tight stable prosthesis even with absence of few screws. However, to provide implant patients with intriguing prospects for rehabilitation with this system, vast research is still required to understand the biomechanical behavior of the OT-Bridge. This investigation aimed to evaluate the stresses induced in an All-on-six maxillary OT-Bridge prosthesis in different circumstances that vary in number and geometric distribution of the retaining screws using finite element analysis.

**Materials & methods:**

This study comprised five test groups based on the number and distribution of the connection screws used to anchor an all-on-six OT-Bridge superstructure as follows; Group I: six screws, Group II: five screws eliminating the right central screw, Group III: four screws with two anterior screws eliminated unilaterally, Group IV: four screws with two anterior screws eliminated bilaterally, and Group V: three screws eliminating both central incisors and right canine screws. The computer simulation was done through three main stages: experimental model designing, SolidWorks modelling, and model analysis using ABAQUS ^®^. The analysis was run under a unilateral axial load (250 N) and an oblique load (150 N). The maximum generated von Mises Stresses (vMS) for the; bridge, prosthetic screws, seeger rings, equators, fixtures, cancellous bone, and cortical bone were recorded to compare the five test groups.

**Results:**

The highest vMS induced in the bridge (677 and 437 MPa under vertical and oblique load, respectively) were in group IV. The highest equator vMS were in groups III and IV (122, 121.9 MPa) under vertical load, while under oblique load, they were in groups V and III (66.2, 65.7 MPa). The vMS in screws, implants, and two bony segments exhibited the most significant increase in group V. The bilateral anterior screw elimination (group IV) compared to the unilateral elimination (group III) increased the vMS on the bridge, screws, and implants.

**Conclusion:**

The OT-Bridge is considered a biomechanically efficient prosthetic system even in the absence of one-third of the anchoring screws in all-on-six prostheses. However, unilateral rather than bilateral elimination of two anterior screws results in better stress distribution pattern.

**Clinical trial number:**

Not applicable.

## Introduction

The reconstruction of the edentulous maxilla with dental implants is a complicated and multifaceted treatment. The maxilla’s anatomical restrictions, high esthetic demand, and atypical resorption pattern present a significant portion of the reconstruction’s difficulty that impacts the ideal implant placement and makes it difficult to design a traditional screw-retained prosthesis; thus, using intermediate abutments is highly advised [1–4]. Thus far, multi-unit abutments (MUA) are the most widely used to convert the implant connections in full arch rehabilitations. They are designed with a range of angle corrections and an external conical connection that allows a passive prosthesis fit. The principal limitations however are related to their wide diameter and weak tiny secondary screw [4, 5]. Recently, the novel low-profile and narrow OT-Equator abutments were proposed as an alternative to the MUA for screw-retained restorations delivered at the abutment level, constituting the central core of the prosthetic project “OT-Bridge” [6, 7]. The OT-Equator has a unique morphology and much more robust structure than MUA’s; it does not need a through screw thus lowering the possibility of fracture, and its locking screw is 30% bigger ensuring more resistance [8, 9].

Owing to its many distinctive components, Rhein 83^®^’s OT-Bridge system was introduced as a creative way to address the shortcomings of the fixed prosthetic systems that were previously available [8]. One of its key components is the sub-equatorial “Seeger”; it is an exchangeable acetal ring housed in a cylindrical “Extragrade” abutment. The Seeger expands to follow the equator head and reverts to its initial shape when it reaches the equator base signifying a monoblock link between the extragrade abutments and the equator which could enable avoiding many unaesthetic screw holes; the clinician will be able to use the abutment in “Blind mode” without anchoring screws retaining the connection only to the seeger, this is a significant feature in the aesthetics zone when implants are not placed in an ideal prosthetic way [9].

Some of the promised benefits of the OT Bridge concept were discussed in the available literature; the biological merits, for instance, are supported by several studies that evaluated the periodontal parameters, clinical outcomes, biological and technical complications, and survival and success rates of this prosthetic project [8, 10–13]. According to Acampora et al. [10], minimal peri-implant bone loss was experienced one year after loading, in agreement with the one-abutment one-time concept [14]. The OT-Equator also provides for the platform-switching concept that aids in minimizing peri-implant bone loss. Moreover, compared to MUA, the lack of locking screws lessens bacterial growth and contamination within the implant [15].

Other advantages of the OT Bridge system supported by Tallarico et al. [9] were related to its ability to overcome high implant divergence, as the Seeger ring gives stability and passivation even with strong disparallelisms [13]. El Mashad et al. [11] also declared that the novel OT-Bridge system, with its integrated seeger, is a promising concept that solves the passivity issue of full arch implant restorations. In addition, the majority of professional CAD software offers the OT-equator as a public library, enabling the prospective planning of prosthetically-driven implant rehabilitations, making it possible to combine well-established surgical methods, like All-on-X, with innovative digital surgical-guided execution of virtual implant planning, ultimately enhancing implant positioning accuracy and immediate loading protocols [16, 17].

One of the most frequent failures and biomechanical complications in implant-supported prostheses is related to screw loosening and fracture [18, 19] The OT-Bridge seeger ring provides a secure elastic retention system of the abutment against the possibility of prosthesis unscrewing [7, 20], this “snap” retention of the extragrade abutment can as well compensate when the initial screw tightening is lost [8, 10]. Moreover, some screws can be avoided without affecting the prosthesis stability; the generic rule is that one of four screws or 25 ٪ of total screws can be avoided [8,9). In this regard, some in-vitro studies demonstrated that the OT-Bridge system could be efficiently used in all-on-four rehabilitations in the absence of one screw among four [16, 20–22]. However, the available literature hasn’t yet investigated the impact of changing the distribution of the eliminated screws on the OT-Bridge biomechanical behavior when more implants are involved in prosthesis support.

Since several variables can affect the stress patterns in the implant-prosthesis-bone complex, finite element analysis (FEA) studies are currently employed to cover significant research gaps on dental implants [23]. Different FEA studies looked into how stress shielding and bone density can affect implant/abutment design and material selection [24–27]. Other studies investigated the impact of implant locations, numbers, and angulations [28–30]. Others focused on the influence of prosthesis materials and design [31, 32]. By simulating the actual behavior of the components under specified load conditions and examining implant/prosthesis lapses relative to the distribution of tensions and deformations within them, these studies will help optimize dental implants and reduce the failure rates for implant therapy.

The potential aesthetic problems that result from labial screw emergence in full-arch prosthesis typically require removing anterior screws. Nonetheless, specific circumstances involving incorrectly angled or positioned posterior or anterior implants during complete arch rehabilitation may require a distinct shift in the allocation of abutments used without screws [9]. This highlights the importance of investigating the distribution of the screwless connections in the maxillary OT-Bridge prostheses. Thus, this FEA study aimed to evaluate the stresses induced in an All-on-six maxillary OT-Bridge prosthesis in different circumstances that vary in number and geometric distribution of the employed retaining screws. The null hypothesis was that; there would be no difference in the peak stress distribution within this prosthetic system between the study groups having different numbers or the study groups having different distributions of connecting screws.

## Materials and methods

**Fig. 1:**
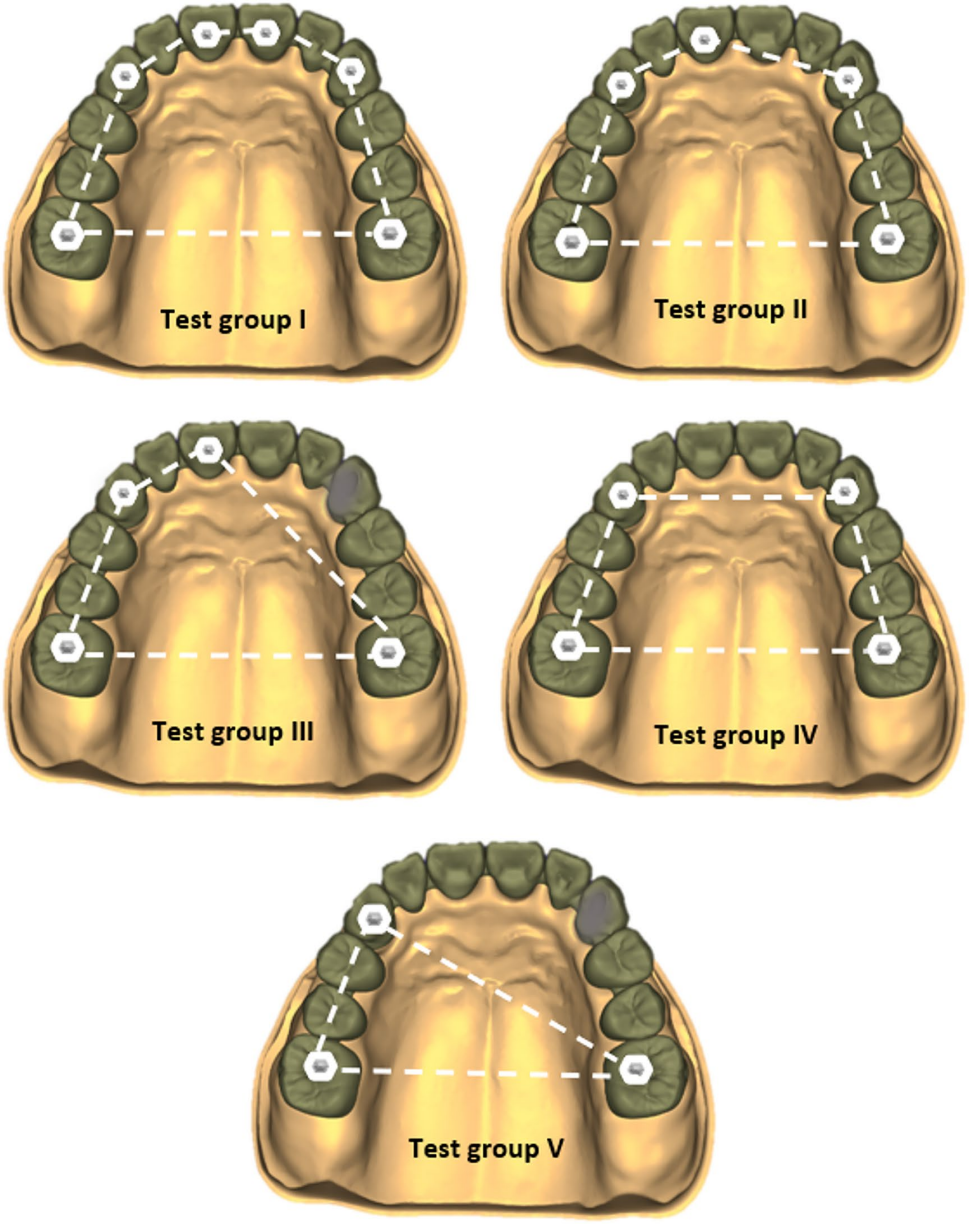
Schematic illustration of the five study groups

This study was conducted on a three-dimensional FE model (3D FEM) of an edentulous maxilla restored with a full arch fixed implant OT-Bridge prosthesis supported by six implants following the Carl Mesh “All-on-six” design [33]. The study compromised five test groups based on the number and geometrical distribution of the connection screws used to anchor the implant superstructure as follows; Group I: Six connection screws were applied, Group II: Five connection screws were applied with elimination of the right central screw, Group III: Four connection screws were applied, entrusting the connection only to the seeger for the right central and canine, Group IV: Four connection screws were applied, entrusting the connection only to the seeger for the two central incisors. Group V: Three connection screws were applied trusting the connection only to the seeger for the two central incisors and the right canine (Fig. 1).

### Designing model geometry and reverse engineering

**Fig. 2:**
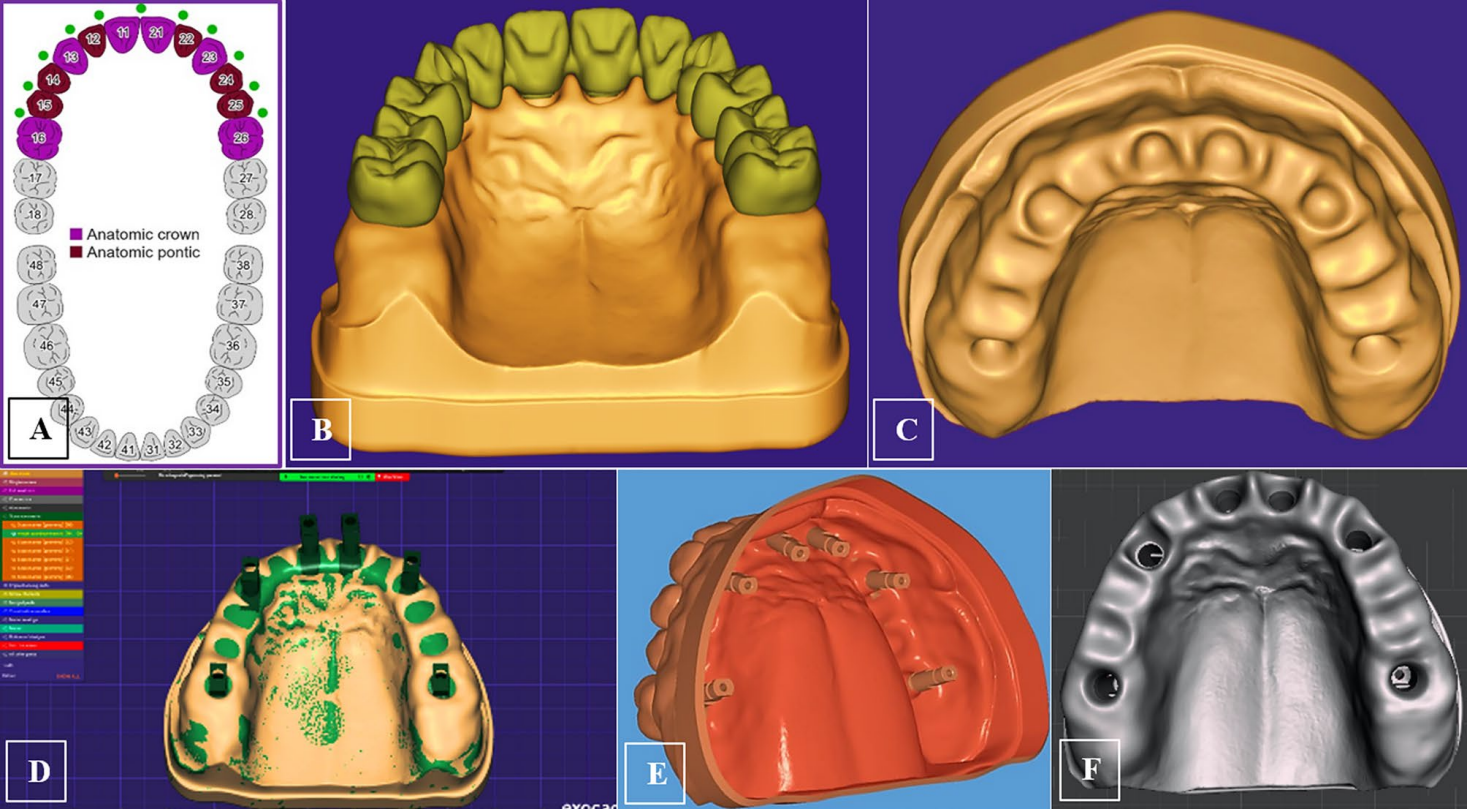
Geometry designing; virtual teeth arrangement over the modeled maxillary arch (**A**,** B**), to define the key implant positions on the model crest (**C**), for positioning the scan bodies (**D**), and implant position detection based on the scan body alignments, (**E**) yielding the final digital implant model design (**F**)

A cone beam computed tomographic (CBCT) scan of an edentulous maxilla that could be rehabilitated with six implants was obtained from the oral radiology department and employed to design the 3D numerical model. The acquired scan data were introduced into the Exocad model-creator module (Exocad GmbH, Hessen, Germany) to create a surface model without noise. A virtual teeth setup was established, and implant position detection was based on six scan bodies (Vitronex Elite Implant System, FLOTECNO SRL, Italy) aligned virtually in six key teeth positions over the model crest (Fig. 2A-D). The matching digital implant analogs from the CAD library were selected and their positions were adjusted within the maxillary model confines as follows; the two anterior implants were located at the central incisors’ positions with 30° angulation, two implants were located at canines’ regions with 16° angulation, and the posterior two implants were situated at the first molars’ region in an upright orientation (Fig. 2E). The digital analogs holes were automatically created in the DIM design based on the final positions and orientation of the analogs, and the soft tissue emergence profile borders were finally defined to complete the model design (Fig. 2F).

**Fig. 3:**
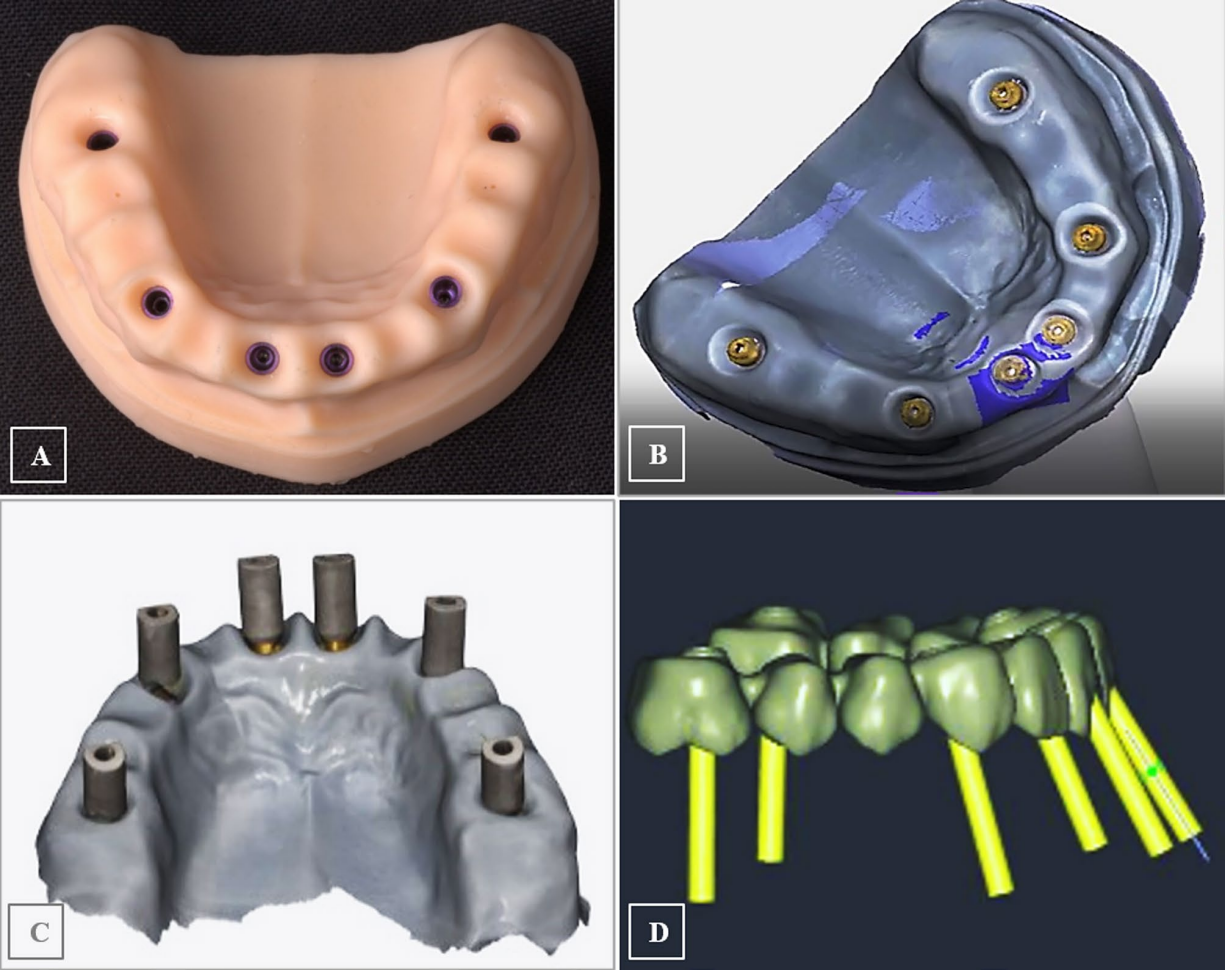
The 3D printed DIM with digital analogs in position (**A**), and model scanning after installation of equator abutment (**B&C**), to generate the design of a twelve-unit OT-bridge based on the replicated implants/equator positions (**D**)

Based on the generated CAM file, the stereolithographic cast was 3D printed (AccuFab-D1s 3D printer, SHINING 3D, China), then six digital analogs were installed in the printed cast (Vitronex, Elite Implant System, Italy) (Fig. 3A). The OT-equator abutments were tightened over the implant analogs (OT Equator abutment, Rhin83, Bologna, Italy), the scan bodies for equator abutments were mounted (Scan body, Rhin83, Bologna, Italy), and the model was scanned using an intraoral scanner (MEDIT I700 intraoral scanner, Seoul, Korea) (Fig. 3B&C). Using the virtual extragrade abutments in the Rhein83 digital library, a single framework was designed as an anatomic setup based on the equator positions, guided by a virtual prosthetic tooth arrangement (twelve teeth from central incisors to first molars bilaterally) (Fig. 3D). The abutment connection of the bridge was modelled, and the seeger rings were simulated inside the extragrade abutments as specified by the manufacturer. A (Seeger-Bridge-Direct) connection from the library was selected to deal with the bridge and abutments as one unit.

**Fig. 4:**
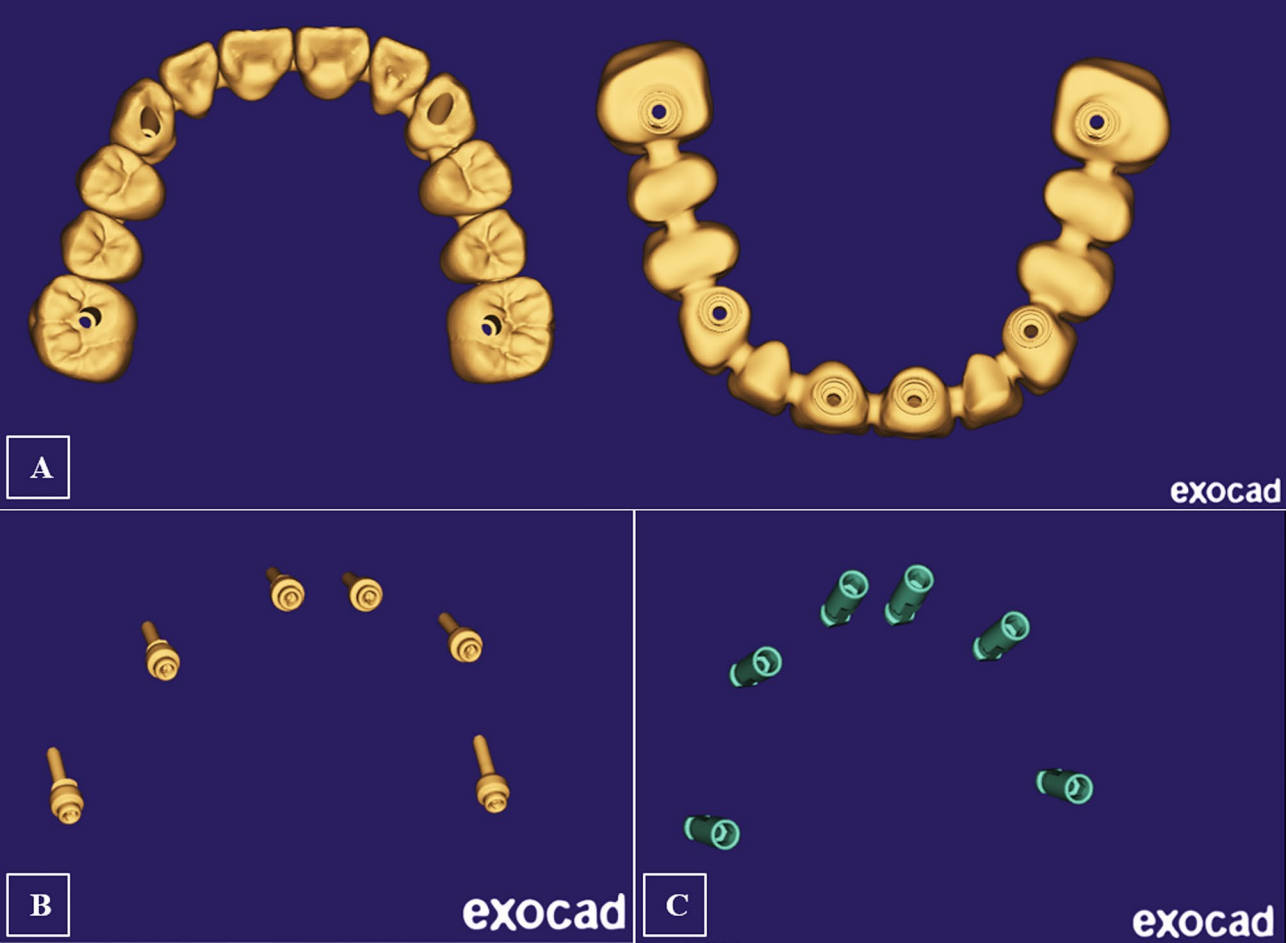
The prepared STL files for the bridge with extragrade abutments (**A**), the orientation of the OT-equators (**B**), and implant analogs (**C**)

All CAD models were converted to Standard Tessellation Language (STL) files. Three STL files were prepared: The bridge with extragrade abutments, the implant analogues, and the OT-equators (Fig. 4A-C). The native STL files were re-meshed to reduce their size while maintaining quality.

### Modeling using solidworks

**Fig. 5:**
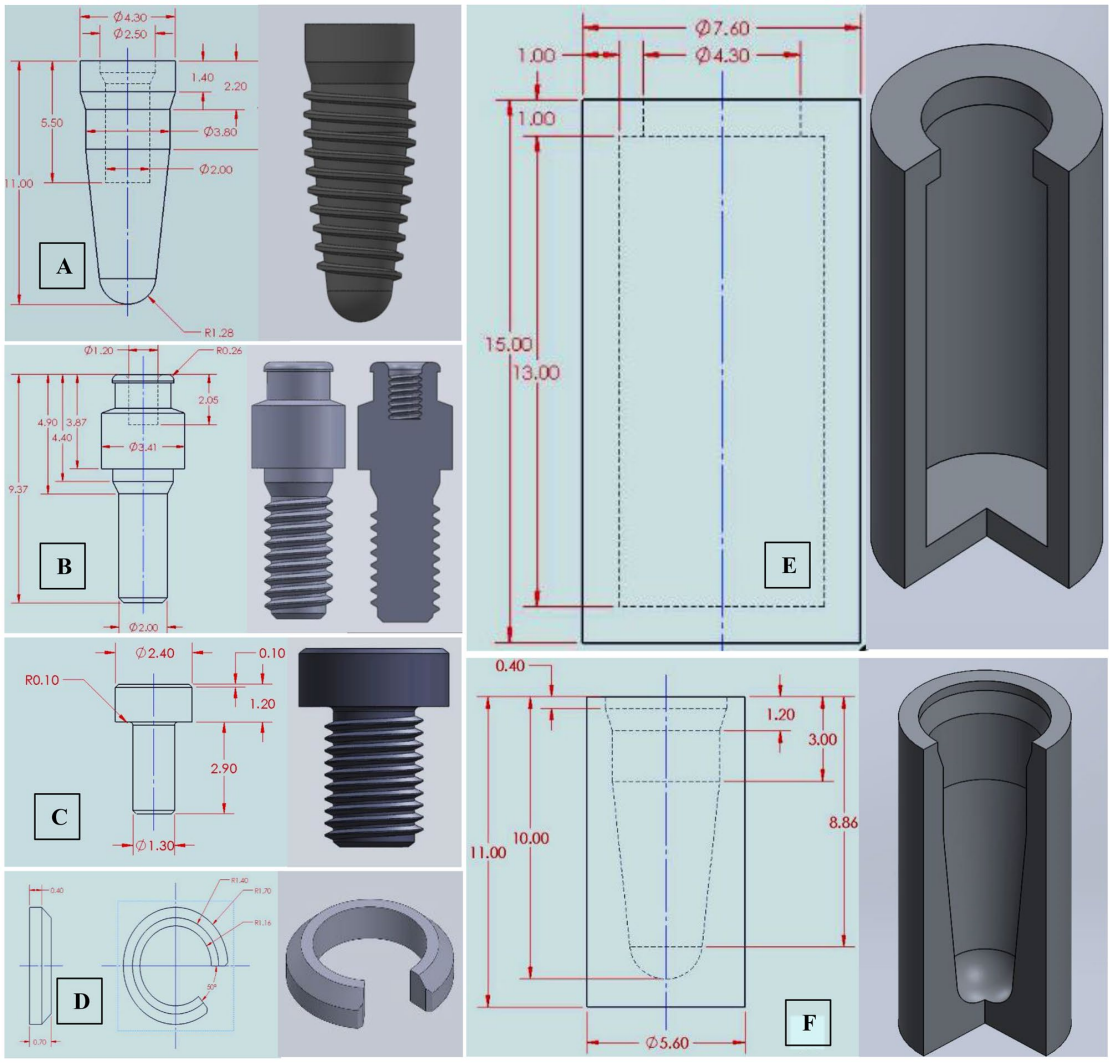
Components’ 2D sketches and their 3D modeling for; implants (**A**), equator abutments (**B**), prosthetic screws (**C**), Seeger rings (**D**), and cortical and cancellous bone cylinders (**E&F**)

Due to the bridge complexity, the 3D information obtained from the STL file was directly used for FE modeling. For implants, screws, and equators, modeling was guided by the corresponding CAD coordinates to replicate their spatial 3D orientation but was accomplished according to accurate dimensions of designs collected from the manufacturer; implants with an internal hex connection and a diameter of 4.3 mm were drawn; measuring 11 mm in length for the four anterior implants and 9 mm for the two posterior implants (CAMLOG Root-Line, Germany), the OT equator missing information was acquired (Rhin83, Bologna, Italy), the equator screw was drawn to be 2 mm in diameter and the prosthetic screw to be 1.3 mm, the seeger was drawn to fill the space between the equator and the bridge intaglio, and the peri-implant bone was schematized as surrounding cylinders; [25, 26, 34] the cortical bone was drawn as 1 mm thick, and the spongy bone was drawn as an internal structure [35]. Finally, all 2D-sketches were converted into 3D-components (Fig. 5A-G).

**Fig. 6:**
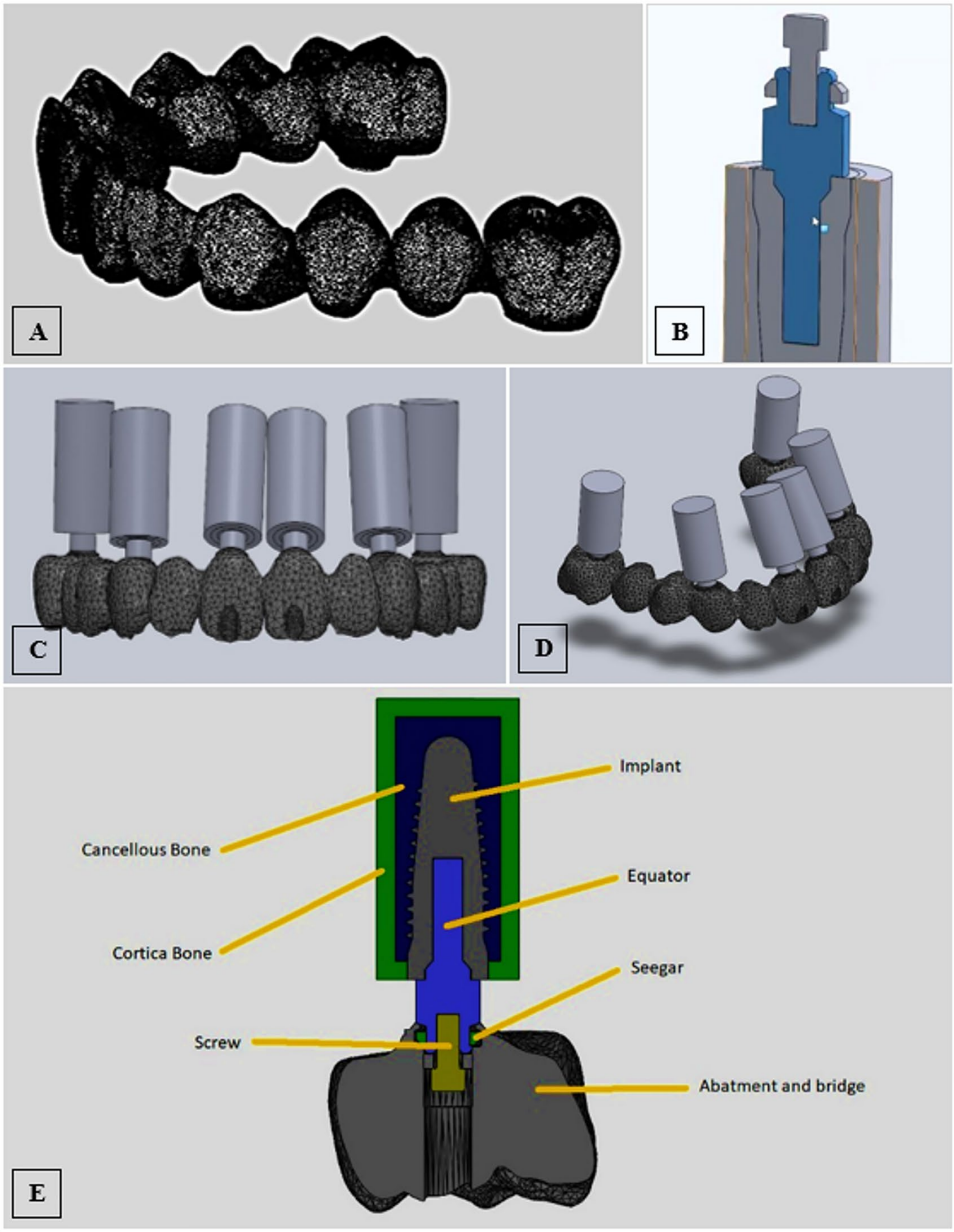
Modeling using SolidWorks; the bridge’s solid body (**A**), a cross-sectional diagram for the repeated-unit subassembly file (**B**), frontal and side views displaying the orientation of the six subassembly files gathered with the bridge STEP file producing the final assembly file (**C**,** D**), and schematic illustration in cross-section for the assembled components (**E**)

The prepared files were converted into a solid body. The assembly module of SolidWorks software (Version 2024, Dassult System, Solidworks Corporation) was used to define the geometry of the assembly; the study components were created as repeated units’ sub-assembly files each composed of an implant surrounded by a cylindrical volume of cortical and cancellous bone with an equator mounted over the implant. The six subassembly files were gathered with the bridge STEP file (standard for the exchange of product data) producing the final assembly file of the bridge supported with the six bony-encompassed implants and connected to them via the OT- equators using prosthetic screws along with six seeger rings allied inside the bridge (Fig. 6A-E).

### Meshing and element type

**Fig. 7:**
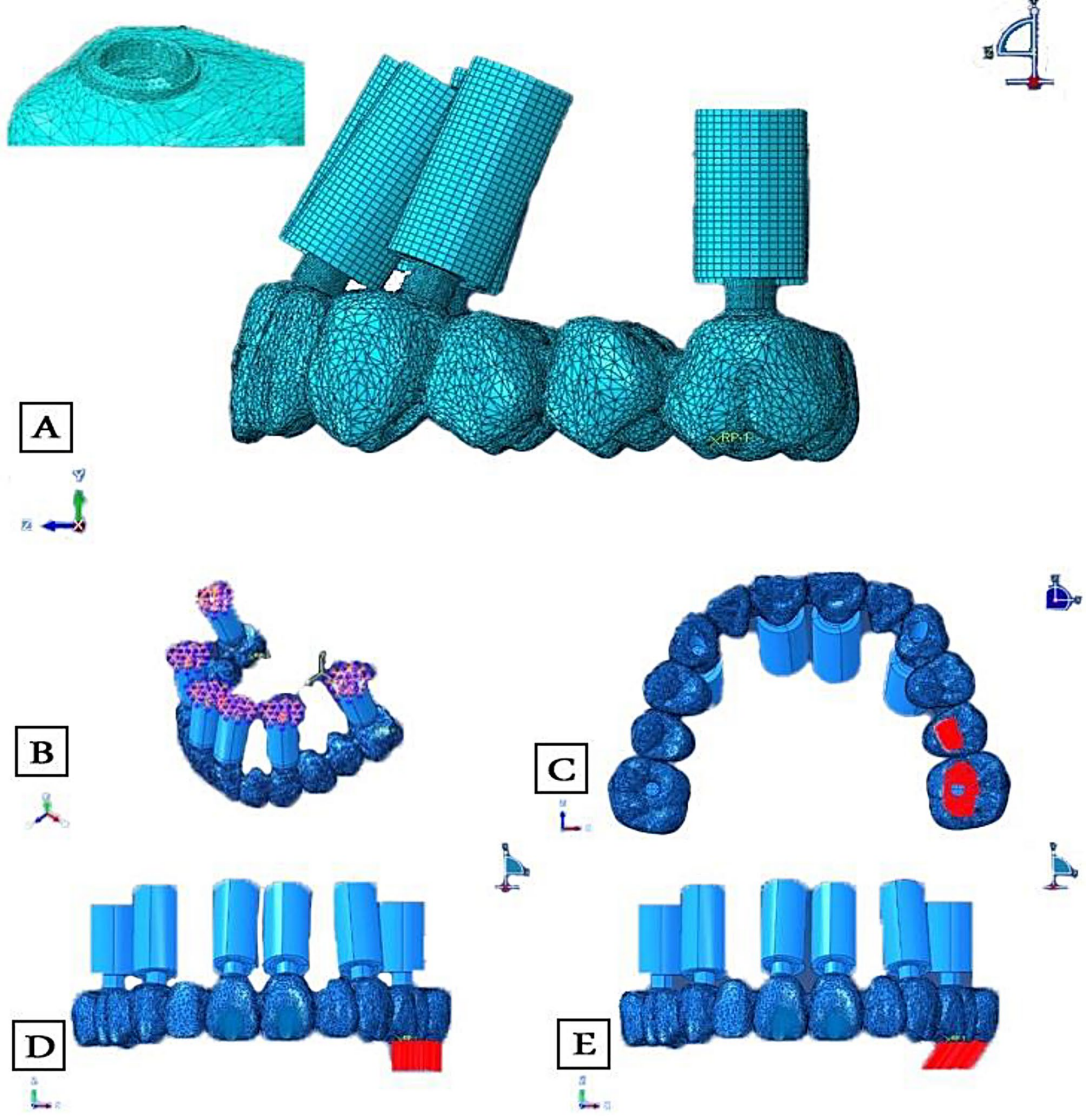
Meshing and analysis using ABAQUS; mesh generation for the components and mesh refining at the connection area (**A**), applying constraints (**B**), and the area of load application on the experimental model (**C**), in vertical (**D**), and oblique directions (**E**)

All files were exported as Parasolid files to be meshed and analyzed in ABAQUS 2024 (Dassault Systèmes Americas Corp., 1301 Atwood Avenue, Suite 101 W, Johnston, RI 02919, USA). The “h-refinement” method was used to perform a mesh convergence; the mesh was refined by increasing the element number from a coarse mesh to a finer mesh until the results converged. The convergence was evaluated by monitoring the changes in the maximum vMS relative to the number of elements. The convergence was met when the relative error in the maximum vMS between two consecutive mesh refinements was below a specified tolerance value (2% in this study) to provide a good balance between accuracy and computational efficiency [26]. Linear hexahedral elements were used for mesh generation, except for meshing of the bridge, modified quadratic tetrahedral elements were adjusted with a 0.5 mm element size, with additional refining to 0.25 mm at equator-bridge connection regions [36] (Fig. 7A). The total element number for the created FEM was 721,137 while the total number of nodes was 1,020,282. The number of nodes and elements of each component are listed in Table 1. The framework, OT equators, screws and implants were simulated to be made of titanium, and the seeger was considered an elastic rubber material [21, 25, 31, 36, 37] All materials were considered isotropic, homogeneous, and linearly elastic. Two essential material parameters were required: elastic (Young’s) modulus (E) and Poisson’s ratio (ν) (Table 2).

**Table 1:** Number of elements and nodes of each component

Component	Element type	Elements	Nodes	Replicates	total elements	total nodes	Average aspect ratio	Worst aspect ratio	Element size (mm)	
1.Compact Bone	C3D8R	740	1,421	4	2,960	5,684	1.69	2.23	0.75	
2.Compact Bone (posterior)	C3D8R	1,272	1,953	2	2,544	3,906	1.55	1.99	0.65	
3.Cancellous Bone	C3D8R	4,040	5,265	4	16,160	21,060	1.49	6.09	0.40	
4.Cancellous Bone (posterior)	C3D8R	2,624	3,511	2	5,248	7,022	1.60	4.87	0.40	
5.Implant (11 mm)	C3D8R	5,880	7,031	4	23,520	28,124	1.74	4.09	0.27	
6.Implant (9 mm)	C3D8R	896	1,208	2	1,792	2,416	2.07	4.01	0.45	
7.Equator	C3D8R	5,822	6,814	6	34,932	40,884	1.84	4.14	0.22	
8.Screw	C3D8R	4,032	4,862	6	24,192	29,172	1.54	3.54	0.14	
9.Seegr	C3D8R	1,070	1,650	6	6,420	9,900	1.66	3.51	0.14	
10.Bridge	C3D10M	603,369	872,114	1	603,369	872,114	1.68	12.07	0.5/0.25 near implant	
Total elements inside the model						721,137				
Total nodes inside the model						1,020,282				

**C3D8R**: linear hexahedral elements

**C3D10M**: Modified quadratic tetrahedral elements

**Table 2:** Defined properties of the materials used

Element	Material	Poisson’s ratio (ν)	Modulus of elasticity (MPa)	Density (tonne/mm3)
Cancellous bone	Bone	0.30	1370 (12)	4.66E-10 (145)
Compact bone	Bone	0.30	13,700 (12)	1.45E-9 (145)
Seeger	elastic	0.35	2600 (134)	1.39E-09 (134)
Implant Equators Framework Screws	Titanium (TiAl6V4)	0.35	110,000 (173)	4.62E-09

Ti indicates; titanium, Al; aluminum, V; vanadium

E: Elastic (Young’s) modulus

### Constraints, interface and boundary conditions

The models were subjected to a rigid fixation restriction in the upper maxilla (Fig. 7B). Before the system equations were ready for solution, they were modified to account for the boundary conditions. All components were constructed with 100% contact along all surface interfaces with no gaps. Perfect integration between bone and implant was considered; this was achieved using a tied contact, so there is no motion between the two structures under applied loading, as recommended by relevant literature [21–23, 31]. The interphase between cortical and cancellous bone layers was considered well-bonded. A friction coefficient was considered 0.3 for the implant/equator and equator/abutment interphases, and 0.35 for the Seeger/equator and Seeger/abutment interphases [21]. Finally, for contact between prosthetic screws and equators, a friction coefficient of 0.5 was defined [34] (Table 3).

**Table 3:** Frictional coefficient and contact type of the components

Connection	Contact type	Friction coefficient
Cancellous bone **/** Compact bone	Bonded	--
Compact-Cancellous bone **/** Implant	Bonded	--
Prosthetic screw **/** Equator	Frictional	0.5
Implant **/** Equator	Frictional	0.3
Equator **/** Seeger	Frictional	0.35
Equator **/** Abutment	Frictional	0.3
Seeger **/** Abutment	Frictional	0.35
Abutment **/** Bridge	Bonded	--

### Loading and running the analysis

**Fig. 8:**
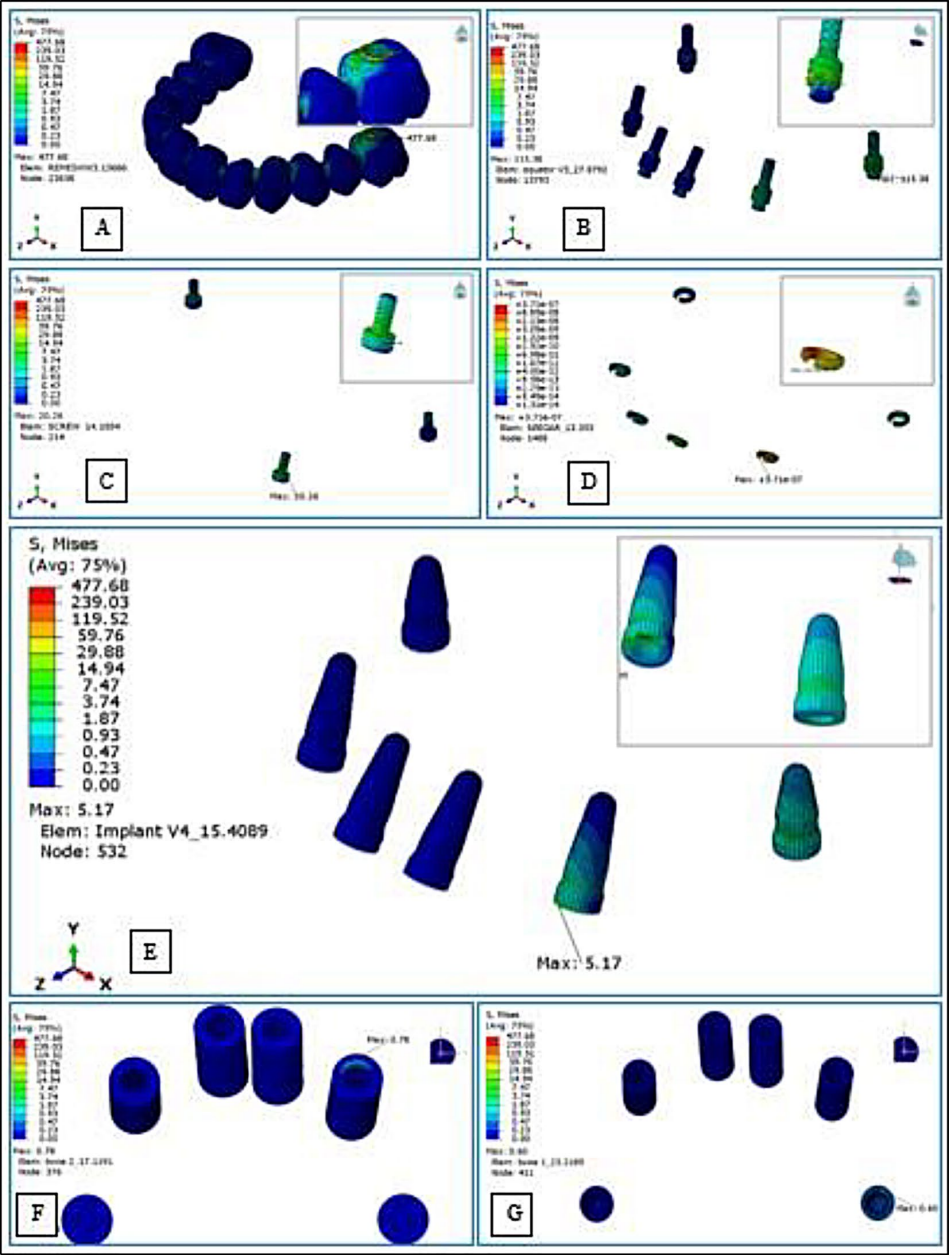
Assessment of von Mises Stresses in; the bridge (**A**), seeger rings (**B**), screws (**C**), equators (**D**), implants (**E**), compact bone (**F**), and cancellous bone (**G**)

Load conditions were applied unilaterally (on the positive Y axis) over an area that included the central fossae of the second premolar and the first molar, where most of the masticatory load is applied in centric occlusion, and it was applied to an area rather than a single point to avoid stress concentration [38]. To mimic the ontogeny of average bite force in humans when rehabilitated by full-arch implant prostheses, the analysis was run under an axial load of 250 N [26, 35, 39], and as oblique forces more accurately reflect occlusal stresses, an oblique load of 150 N was applied to the same sites at 30° inclination in a palato-buccal direction [31, 34] (Fig. 7C-E). Dynamic analysis was accomplished, and the maximum generated vMS in the; bridge, prosthetic screws, seeger rings, equators, fixtures, cortical, and cancellous bone were calculated (Fig. 8A-G).

### Statistical analysis

The vMS values were recorded and tabulated for the five study groups under the two load conditions (Table 4). Due to the nature of FEA, there was no need to calculate the sample size because it is a computer simulation where all co-founders can be controlled. Upon repeating the test many times, the same result will be achieved, thus, it was not feasible to conduct a comprehensive statistical analysis for the resultant stresses [40].

**Table 4:** vMS values (MPa) and peak-stress concentration areas in the components under vertical and oblique loads

	Group I		Group II		Group III		Group IV		Group V	
	vertical	Oblique	vertical	oblique	Vertical	oblique	vertical	oblique	vertical	oblique
Bridge	654.3 left molar	360 left molar	654.3 left molar	361 left molar	651.4 Left molar	360 left molar	677 left molar	437 left molar	478 left molar	201.5 left molar
Screw	16.4 left canine	14.3 left canine	17 Left canine	17.6 left canine	17 Left canine	19.1 left canine	18 Left canine	21.2 left canine	20.3 Left canine	88.7 Left canine
Seeger	4 ^e-7^ Left canine	2 ^e-9^ left canine	2 ^e-8^ Left central	1 ^e-8^ left canine	8 ^e-8^ Left central	2 ^e-8^ Left central	1 ^e-4^ Right canine	5 ^e-9^ Right canine	1 ^e-9^ Left canine	3 ^e-6^ Right central
Equator	121 Left molar	63 Left molar	121 Left molar	63 Left molar	122 Left molar	65.7 Left molar	121.9 Left molar	55.4 Left molar	115 Left molar	66.2 Left molar
Implant	3.4 Left canine	4.6 Left molar	3.4 Left canine	4.8 Left molar	3.4 Left canine	5.1 Left central	4.1 Left canine	5.5 Left canine	5.2 Left canine	9.9 Left molar
Compact bone	0.52 Left canine	0.83 Left canine	0.52 Left canine	0.88 Left molar	0.52 Left canine	0.9 Left molar	0.61 Left canine	0.9 Left molar	0.8 Left canine	1.0 Left molar
Cancellous bone	0.56 Left molar	0.39 Left molar	0.56 Left molar	0.39 Left molar	0.56 Left molar	0.4 Left molar	0.57 Left molar	0.39 Left molar	0.6 Left molar	0.7 Left molar

## Results

**Fig. 9:**
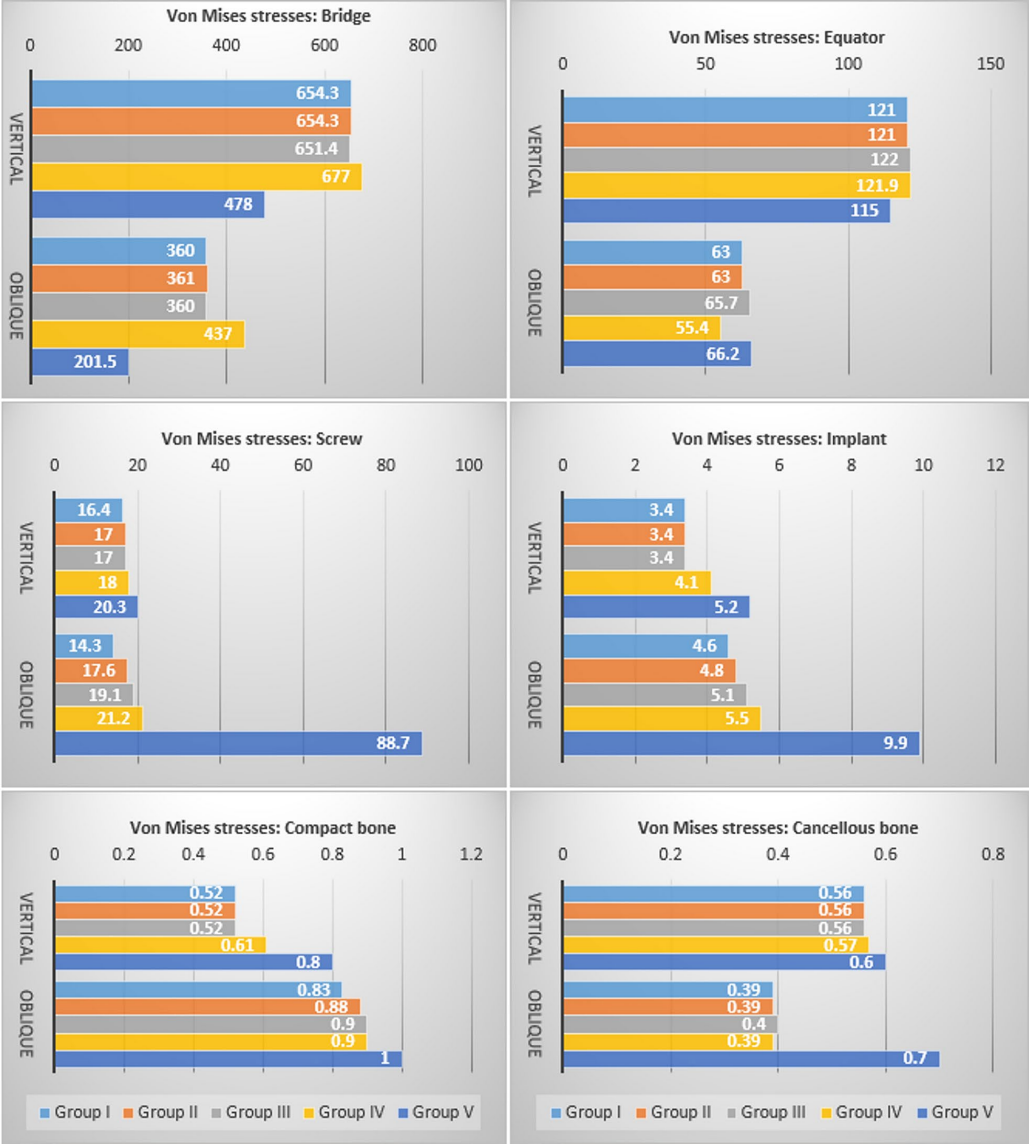
Descriptive charts for comparison of von Mises Stress between the groups: bridge, equators, screws, implants, compact, and cancellous bone under vertical and oblique load

In all groups, the highest stress values were recorded for the bridge followed by the equator abutments, screws, implants, and bony segments respectively, and the lowest negligible stress values were recorded for the seeger. All groups demonstrated similar stress distribution patterns, marked by gradual stress dispersion from the load-bearing to the non-load-bearing sides, except for group V, which exhibited more concentrated stresses on the load-bearing side. The peak of all component stresses was mostly concentrated at the first molar region where the load was applied, however, the stresses were greatest at the left canine region for the screws under both loading conditions, and for the implants and the compact bone under vertical loading (Table 4). Due to the limited sample size of one for each group, descriptive statistics were retrieved from the analyzed FEMs to compare the vMS values for each component between the groups and between vertical and oblique loading conditions (Fig. 9).

### Intergroup comparisons of induced stresses in the different components

For the bridge, the highest stress values under both load conditions were found in group IV, while the lowest values were in group V. For the OT equator, the highest value under axial load was in group III, while the lowest was in group V. On the other hand, the highest value under oblique load was recorded for group V, while the lowest was recorded for group IV. With the elimination of more than one-third of the screws in group V, the stresses on the screws, implants, and the two bone segments significantly increased. Under both load conditions, the most stressed screws were found in group V and group I screws were the least stressed (the stresses on the screws were 4 times higher under oblique load). The highest stress values recorded for implants and peri-implant bone were also in group V, while the lowest was in group I (implants and cancellous bone showed nearly a twofold increase in stresses under oblique load). Regarding the geometric distribution of the eliminated screws, the results displayed that the bilateral elimination of the two anterior screws in group IV compared to the unilateral screw elimination in group III increased the stresses on the bridge by 1.04 and 1.21 times, on the screws by 1.05 and 1.1 times, and on the implants by 1.2 and 1.07 times under vertical and oblique loads respectively (Fig. 9).

### Intragroup comparisons of induced stresses under vertical and oblique loading

von Mises Stress values recorded for the bridge and the equator were higher under vertical load than under oblique load in all groups; the peak stress at the equators under vertical load was nearly twice that recorded under oblique load. However, the peak stresses for screws were slightly higher under oblique load than that recorded under vertical load particularly in group V; there was a massive increase in stresses under oblique load compared to those recorded under vertical load. However, in group I, the stresses were higher under vertical load. The peak stresses for the implants and cortical bone under vertical load were lower than those recorded under oblique load. For the cancellous bone, only in group V the stresses induced under oblique load were greater than vertical load, in contrast to the trend observed in all other groups, where stresses induced under vertical load were greater than under oblique load (Fig. 9).

## Discussion

In the present study, the null hypothesis was rejected as the results showed that the peak stresses induced in the implant-retained OT-Bridge were affected by the number of eliminated screws, as well as their geometric distribution, where the elimination of more than two screws out of six generated different stress patterns compared to other designs, and also bilateral elimination of two anterior screws generated higher stresses than their unilateral elimination.

Some studies demonstrated the reliability of the OT-Bridge in all-on-four mandibular rehabilitation when one of the screws is absent [16, 21, 22]. However, in the current study, the five clinical situations were simulated for a maxillary model restored with six implants to reflect the esthetic demand for maxillary rehabilitations [41] because no studies provided precise guidelines for screwless abutment use when more than four implants are used to support a maxillary prosthesis. Long-span situations also exhibit increased stresses and reduced survival prediction than short-span cases, thus, the prosthetic structure is more critical for preserving the system stability [42, 43].

The results of our study have been compared for validity with other experimental studies in the literature [16, 21, 22, 31]; one was a FEA study similar to our work, conducted to investigate the mechanical behavior of the OT-Bridge in a mandibular model with four implants. However, in this study [21], the prosthesis was subjected to 800 N load for test groups (1&2) and 700 N load for test groups (3&4) divided into the two molar regions, although these force levels might not hold in reality, the authors used a failure criterion where simulations were terminated upon reaching a specific displacement values, and comparisons were made based on the equivalent load that caused failure for each group. In contrast to our study, using a constant, realistic load across all groups allows the portrayal of a real clinical state and evaluation of the structural performance under standardized loading conditions.

The vMS criterion is widely used to assess stress in metals and other ductile materials [32, 37]. The highest vMS values were recorded for the bridge followed by the equator abutments, screws, implants, and bony segments respectively. These findings corroborate with other studies [21, 31, 44, 45] and indicate favorable biomechanical behavior of the OT-bridge prosthesis in all groups regardless of the number of eliminated screws, as in the same context the forces are absorbed by the rigid superstructure transmitting lower vMS in implants and bone [46].

The stress values observed in all test groups were within the physiological limits of bone tissue as according to ultimate bone strength, overloading in cortical bone occurs when the σ min exceeds 170 to 190 MPa and, the σ max exceeds 100 to 130 MPa, while overloading in trabecular bone happens when either σ min or σ max exceeds 5 MPa [47, 48]. The reduced bone stresses observed in the current study can be correlated to the OT-equator’s unique design that can concentrate tension over the retainer’s head, allowing minor stresses to distribute across the fixture and peri-implant bone [6]. Furthermore, a portion of the stresses could be dissipated by the seeger ring elastic action [16, 21, 22].

Although the highest stresses were recorded in the prosthetic superstructure, all stress values recorded for all groups were within the tensile strength limit of titanium (860 to 965 MPa) [49]. On the other hand, the lowest stress values were recorded for the seeger, which could be explained by the fact that it is made of acetal resin and has a distinctive open-ring design. Acetal resins are considered bridge-building polymers between plastic and metal; they provide the durability of metal and the comfort and flexibility of plastics, and they are characterized by having a strong elastic memory [50]. In a state of tension, the seeger rings can distort themselves, dissipating the stresses due to their strong elastic memory and incomplete circumference [16].

It is evident from the current study results that groups; I, II, and III had nearly identical peak stress distribution patterns in the system; they demonstrated a more balanced biomechanical performance with less stress concentration on the implant and bone compared to groups IV and V. This highlights that using five or even four prosthetic screws instead of six still seems to be safe and predictable, certifying the seeger ring’s ability to interlock the prosthesis and counterbalance the imposed forces [22]. These findings are consistent with the results of Cervino’s and Milone’s studies [16, 21].

Based on the analysis of the current study findings, removing three screws out of six in group V dramatically changed the force distribution pattern. Although the highest stresses recorded for the bridge and equator abutments were in groups III and IV rather than group V as was expected when a greater portion of the framework becomes a cantilever without screws [46], the stresses on the screws, the implants, and the two bony segments showed the expected ascending increase with the elimination of more screws from group I to group V which align with other study findings where removing connection screws increased stress distribution to bone [21]. In group V, the reduced stresses in the bridge under both load conditions and in equators under vertical load were accompanied by significantly higher stress concentrations in the screws, implants, and compact and cancellous bone, indicating less favorable biomechanical behavior, greater risk of clinical complications such as; screw loosening and loss of osseointegration in an equivalent real-life scenario if half of the screws were discarded.

Regarding the effect of the geometric distribution represented by the different positions of the two eliminated screws in groups III and IV, the results displayed that the bilateral elimination of the two anterior screws in group IV compared to the unilateral screw elimination in group III increased the stresses on bridge, screws, implants, and bone, this validates the speculation that the prosthesis could behave differently when the geometric distribution of the removed screws changes. Compared to group III, group IV recorded higher vMS in the bridge, but lower stress concentration in equator abutments, the quadrilateral spread-out of the applied screws that tightly connect the superstructure to the implants could explain higher force absorption by the bridge [51, 52]. Group IV simultaneously showed higher stress levels in the bone, implants, and screws thus demonstrating less advantageous biomechanical behavior; according to Benzing’s study [51], this means increased prosthesis deformation near the load application.

A FEA study conducted on six implant-supported frameworks with different cantilever lengths demonstrated that when the framework acts as a cantilever, the longer the lever arm, the higher the stresses [46]. Similarly, we can interpret the findings of the current study; the disparity between groups III and IV in the surface area covered by the connecting screws versus the cantilevered area retained only by the seeger could be the reason behind the redistribution of the stresses although same screw count was applied; the increased frontal cantilever length in group IV relative to the anchored area seems to be a logical interpretation [33, 46, 51].

Regarding peak stress concentration patterns, all five study groups showed similar results, the first molar region was primarily where the peak of each component stress was located regardless of the position of the eliminated screws, this finding was consistent with several studies that demonstrated the highest stress concentration points coincided with the location of the load application [21, 31, 32, 52]. However, the stresses were greatest at the left canine screws under both loading conditions, and at the left canine implants and compact bone under vertical loading. These findings could be related to the stress absorption by the bridge and abutments in the premolar-molar region, so stresses are transmitted more to infrastructure as we get farther from the location of the load application, and reach its peak at the nearest canine due to its location at the corner of the arch [53]. This might signify how crucial it is to carefully choose the occlusal scheme relative to the locations of the removed screws in the OT-bridge prosthesis [54]. On the positive side, the screws in every group had stress values that were less than titanium’s tensile and compressive strengths, preventing an instantaneous fracture [55].

The peak stress distribution within the seeger rings varied between the groups; in groups I and II, the highest seeger stresses were recorded at the left canine region; however, stress peaks were shifted to the left central position in group III and to the right canine position in group IV. In group V, the greatest seeger stresses under vertical load were at the left canine position, but under oblique load at the right central incisor. A recent investigation that assessed the forces generated at the seeger level during loading demonstrated comparable findings; they concluded that the seeger rings closer to the screwless abutment and load application, and seeger rings of screwless connections were the most supposed to deform because of the intensity of force generated on the XY plane and the Z-axis [22].

Providing the current literature with these in vitro testing outcomes would help clinicians comprehend the clinical implications of determining the most appropriate prosthetic design when rehabilitating their patients using the OT-Bridge system. However, the study’s findings may be influenced by the additional in vivo biological and clinical complexities, thus, they should be regarded cautiously. Another limitation of this study is related to the employed static loading conditions, which might underestimate the loading effect in the case of dental implants and fail to account for the potential reasons for fatigue failures or fractures [23, 43]. Furthermore, completing the simulation typically requires making certain assumptions that are standard in FEA studies but might not hold in the real situation [56]. However, due to the comparative nature of the current study, such assumptions wouldn’t interfere with the aim because they exist in all the groups.

Considering these points, future FEA studies simulating the dynamic nature of forces that occur during chewing and the other implant-supported treatment alternatives for maxilla and mandible that include; different implant numbers, different geometric distribution of eliminated screws, different framework materials and designs, and different occlusal designs are needed to verify the findings of this study. Also, clinical trials with long-term follow-up are necessary to confirm the relevance of these findings in clinical cases.

## Conclusions

Within the limitations of the current study, the following could be concluded:

- The OT-Bridge is considered a biomechanically efficient prosthetic system even in the absence of one-third of the anchoring screws in all-on-six prostheses. Nevertheless, eliminating more than two screws out of six increases the stresses generated in the prosthetic components and the peri-implant environment.
- The number of eliminated screws, as well as their geometric distribution, influence the stress pattern in the OT-Bridge prostheses; for all-on-six prostheses, unilateral rather than bilateral elimination of two anterior screws results in better biomechanical behavior.

## Data Availability

The datasets used and/or analyzed during the current study are available from the corresponding author upon reasonable request.

## Declarations

### Study protocol reporting

The study protocol has been posted on Protocol Exchange, an open repository of community-contributed protocols sponsored by Nature Portfolio and was made freely available to the scientific community for use and comment on 2/2/2024. This work is available at: 10.21203/rs.3.pex-2549/v1.

## Abbreviations

CAD/CAM: Computer-Aided Design and Computer-Aided Manufacturing
vMS: von Mises Stresses
MPa: Mega Pascal
MUA: Multi-unit abutments
CBCT: Cone Beam Computed Tomography
FE: Finite element
FEA: Finite element analysis
FEM: Finite element model
IOS: Intraoral scanner
2D: Two-dimensional
3D: Three-dimensional
DIM: Digital implant model
STL: Standard Tessellation Language
STEP: Standard for the exchange of product data
E: Elastic (Young’s) modulus
ν: Poisson’s ratio
mm: Millimeter
N: Newton
tonne/mm3: Tonne per cubic millimeter
C3D8R: linear hexahedral elements
C3D10M: Modified quadratic tetrahedral elements

## References

[1] Abdunabi A, Morris M, Nader SA, Souza RF. Impact of immediately loaded implant-supported maxillary full-arch dental prostheses: a systematic review. J Appl Oral Sci. 2019;27:e20180600. 31411262 10.1590/1678-7757-2018-0600 PMC9648956

[2] Gallucci GO, Avrampou M, Taylor JC, Elpers J, Thalji G, Cooper LF. Maxillary implant-supported fixed prosthesis: a survey of reviews and key variables for treatment planning. Int J Oral Maxillofac Implants. 2016;31:s192–197. 27228251 10.11607/jomi.16suppl.g5.3

[3] Ashurko I, Trofimov A, Tarasenko S, Mekhtieva S. Full-Mouth Screw-Retained Implant-Supported rehabilitation with multiunit abutments using virtual guided surgery and digital prosthetics protocol. Case Rep Dent. 2020;2020:3585169. 32963837 10.1155/2020/3585169 PMC7501563

[4] Kher U, Tunkiwala A, Patil PG. Management of unfavorable implant positions and angulations in edentulous maxillae with different complete-arch fixed prosthetic designs: A case series and clinical guidelines. J Prosthet Dent. 2022;127(1):6–14. 33243475 10.1016/j.prosdent.2020.09.023

[5] Janev EJ, Redzep E, Janeva N, Mindova S. Multi-unit abutments recommended in prosthetic and surgical implantology treatment (case report). J Morphological Sci. 2020;3(1):65–72.

[6] Grande F, Cesare PM, Zamperoli EM, Gianoli CM, Mollica F, Catapano S. Evaluation of tension and deformation in a mandibular Toronto Bridge anchored on three fixtures using different framework materials, abutment systems, and loading conditions: A FEM analysis. Eur J Dent. 2023;17:1097–105. 36696917 10.1055/s-0042-1758785 PMC10756777

[7] Catapano S, Ferrari M, Mobilio N, Montanari M, Corsalini M, Grande F. Comparative analysis of the stability of prosthetic screws under Cyclic loading in implant prosthodontics: an in vitro study. Appl Sci. 2021;11(2):622.

[8] Montanari M, Scrascia R, Cervino G, Pasi M, Ferrari E, Xhanari E, Koshovari A, Tallarico M. A one-year, multicenter, retrospective evaluation of narrow and low-profile abutments used to rehabilitate complete edentulous lower arches: the OT Bridge concept. J Prosthodont. 2020;2:352–61.

[9] Tallarico M, Scrascia R, Annucci M, Meloni SM, Lumbau AI, Koshovari A. Errors in implant positioning due to lack of planning: a clinical case report of new prosthetic materials and solutions. Materials. 2020;13(8):1883. 32316361 10.3390/ma13081883 PMC7215328

[10] Acampora R, Montanari M, Scrascia R, Ferrari E, Pasi M, Cervino G. 1-Year evaluation of OT Bridge abutments for immediately loaded maxillary fixed restorations: a multicenter study. Eur J Dentistry. 2020;15(02):290–4. 10.1055/s-0040-1716632 PMC8195625 33622005

[11] El Mashad TG, Fayyad AE, Awaad N. Comparison between bone height changes around the splinted and non-splinted OT Bridge system implants in edentulous mandibles rehabilitated with all-on four full arch prosthesis a randomized clinical trial. Int J Health Sci. 2022;6(S10):214–24.

[12] Fayyad AE. Prosthetic complications in immediately loaded All-on-Four mandibular prosthesis using titanium wire reinforcement versus non-reinforced temporary framework. A randomized clinical trial. Egypt Dent J January. 2022;68(1):885–92.

[13] Gomaa AS, Dehis WM, Mohamad OA, Ali EH, Khalil DK. Radiographic evaluation of crestal bone loss around implants in mandibular all on 4 implant hybrid prosthesis using 2 different abutments (Multi-unit versus OT Bridge). Egypt Dent J Oct. 2024;70(4):3549–59.

[14] Ríos-Santos JV, Tello-González G, Lázaro-Calvo P, Gil Mur FJ, Ríos-Carrasco B, Fernández-Palacín A, Herrero-Climent M. One abutment one time: A multicenter, prospective, controlled, randomized study. Int J Environ Res Public Health. 2020;17(24):9453. 33348644 10.3390/ijerph17249453 PMC7765846

[15] Tallarico M, Canullo L, Caneva M, Özcan M. Microbial colonization at the implant-abutment interface and its possible influence on periimplantitis: A systematic review and meta-analysis. J Prosthodontic Res. 2017;61(3):233–41. 10.1016/j.jpor.2017.03.001 28359872

[16] Cervino G, Cicciu M, Fedi S, Milone D, Fiorillo L. FEM analysis applied to OT Bridge abutment system with seeger retention system. Eur J Dent. 2021;15(01):47–53. 32869222 10.1055/s-0040-1715550 PMC7902099

[17] Piscopo M, Grande F, Catapano S. Full digital workflow for prosthetic Full-Arch immediate loading rehabilitation using OT-Bridge system: A case report. Prosthesis. 2022;4:213–23.

[18] Pardal-Peláez B, Montero J. Preload loss of abutment screws after dynamic fatigue in single implant-supported restorations. A systematic review. J Clin Exp Dent. 2017;9(11):e1355–61. 29302289 10.4317/jced.54374 PMC5741850

[19] Goodacre CJ, Bernal G, Rungcharassaeng K, Kan JY. Clinical complications with implants and implant prostheses. J Prosthet Dent. 2003;90(2):121–32. 12886205 10.1016/S0022-3913(03)00212-9

[20] Pozzan MC, Grande F, Mochi Zamperoli E, Tesini F, Carossa M, Catapano S. Assessment of preload loss after Cyclic loading in the OT Bridge system in an All-on-Four rehabilitation model in the absence of one and two prosthesis screws. Mater (Basel). 2022;15(4):1582. 10.3390/ma15041582 PMC8879715 35208121

[21] Milone D, Nicita F, Cervino G, Santonocito D, Risitano G. Finite element analysis of OT Bridge fixed prosthesis system. Procedia Struct Integr. 2021;33:734–47.

[22] Grande F, Pozzan MC, Marconato R, Mollica F, Catapano S. Evaluation of load distribution in a mandibular model with four implants depending on the number of prosthetic screws used for OT-Bridge system: A finite element analysis (FEA). Mater (Basel). 2022;10(22):7963. 10.3390/ma15227963 PMC9699052 36431449

[23] Falcinelli C, Valente F, Vasta M, Traini T. Finite element analysis in implant dentistry: state of the Art and future directions. Dent Mater. 2023;39:539–56. 37080880 10.1016/j.dental.2023.04.002

[24] Ouldyerou A, Aminallah L, Merdji A, Mehboob A, Mehboob H. Finite element analyses of porous dental implant designs based on 3D printing concept to evaluate Biomechanical behaviors of healthy and osteoporotic bones. Mech Adv Mater Struct. 2022;30(11):2328–40.

[25] Rajaeirad M, Fakharifar A, Posti MHZ, Khorsandi M, Watts DC, Elraggal A, Ouldyerou A, Merdji A, Roy S. Evaluating the effect of functionally graded materials on bone remodeling around dental implants. Dent Mater. 2024;40(5):858–68. 38616152 10.1016/j.dental.2024.04.002

[26] Mehboob H, Ouldyerou A, Ijaz MF. Biomechanical investigation of Patient-Specific porous dental implants: A finite element study. Appl Sci. 2023;13(12):7097.

[27] Ouldyerou A, Merdji A, Aminallah L, Mehboob H, Mehboob A, Roy S, Goswami T, Mukdadi OM, Tarlochan F. Functionally graded ceramics (FGC) dental abutment with implant-supported cantilever crown: finite element analysis. Compos Commun. 2023;38:101514. 10.1016/j.coco.2023.101514

[28] Tezerişener HA, Özalp Ö, Altay MA, Sindel A. Comparison of stress distribution around all-on-four implants of different angulations and zygoma implants: a 7-model finite element analysis. BMC Oral Health. 2024;24(1):176. 38310260 10.1186/s12903-023-03761-x PMC10837953

[29] Lee H, Jo M, Sailer I, Noh G. Effects of implant diameter, implant-abutment connection type, and bone density on the Biomechanical stability of implant components and bone: a finite element analysis study. J Prosthet Dent. 2022;128(4):716–28. 33685654 10.1016/j.prosdent.2020.08.042

[30] Shen L, Dong C, Chen J, Bai X, Yang F, Wang L. The mechanical and clinical influences of prosthetic index structure in Morse taper implant-abutment connection: a scoping review. BMC Oral Health. 2023;23(1):775. 37865734 10.1186/s12903-023-03545-3 PMC10590505

[31] Bhering CL, Mesquita MF, Kemmoku DT, Noritomi PY, Consani RL, Barão VA. Comparison between all-on-four and all-on-six treatment concepts and framework material on stress distribution in atrophic maxilla: A prototyping guided 3D-FEA study. Mater Sci Eng C Mater Biol Appl. 2016;69:715–25. 27612765 10.1016/j.msec.2016.07.059

[32] Kupprano P, Kamonkhantikul K, Homsiang W, Arksornnukit M, Takahashi H. Finite element analysis on implant-supported bar with different geometric shapes. BMC Oral Health. 2024;24(1):1572. 39736666 10.1186/s12903-024-05373-5 PMC11684229

[33] Misch CE. Dental implant Prosthetics-E-Book. St. Louis: Elsevier Health Sciences; 2014.

[34] Cicciù M, Cervino G, Milone D, Risitano G. FEM analysis of dental implant-abutment interface overdenture components and parametric evaluation of Equator^®^ and Locator^®^ prosthodontics attachments. Materials. 2019;12(4):592. 30781478 10.3390/ma12040592 PMC6416601

[35] Youssef SA, Galal RM, Aldhahri GFS, Alzaylaei RA, Alamoudi MSM, Benjabi AAA, Jayar YH, Alsaiary AMM, Aljuhani AN, Sanyour AOA, Khoja AAA, Sanyour AOA, Khamis AMM, Alghalayinib SS. Finite element analysis on all on four implant screw retained mandibular fixed prosthesis. (Material selection role). Int J Innovative Res Med Sci. 2020;5(12):650–5.

[36] Byun SH, Seo JH, Cho RY, Yi SM, Kim LK, Han HS. Finite element analysis of a new Non-Engaging abutment system for Three-Unit Implant-Supported fixed dental prostheses. Bioengineering. 2022;9(10):483. 36290451 10.3390/bioengineering9100483 PMC9598935

[37] Ibrahim CRM, Sameh A, Askar O. A finite element analysis study on different angle correction designs for inclined implants in All-On-Four protocol. BMC Oral Health. 2024;24(1):331. 38481220 10.1186/s12903-024-04091-2 PMC10938696

[38] Rees JS. An investigation into the importance of the periodontal ligament and alveolar bone as supporting structures in finite element studies. J Rehabil. 2001;28(5):425–32. 10.1046/j.1365-2842.2001.00686.x 11380782

[39] Topcu Ersöz MB, Mumcu E. Biomechanical investigation of maxillary implant-supported full-arch prostheses produced with different framework materials: a finite elements study. J Adv Prosthodont. 2022;14(6):346–59. 36685790 10.4047/jap.2022.14.6.346 PMC9832146

[40] Aboelfadl A, Keilig L, Ebeid K, Ahmed MAM, Nouh I, Refaie A, Bourauel C. Biomechanical behavior of implant retained prostheses in the posterior maxilla using different materials: a finite element study. BMC Oral Health. 2024;24(1):455. 38622680 10.1186/s12903-024-04142-8 PMC11020654

[41] Bidra AS. Three-dimensional esthetic analysis in treatment planning for implant‐supported fixed prosthesis in the edentulous maxilla: review of the esthetics literature. J Esthetic Restor Dentistry: Official Publication Am Acad Esthetic Dentistry. 2011;23(4):219–36. 10.1111/j.1708-8240.2011.00428.x 21806753

[42] Bryant SR, MacDonald-Jankowski D, Kim K. Does the type of implant prosthesis affect outcomes for the completely edentulous arch? Int J Oral Maxillofacial Implants. 2007;22:117–39. 18437794

[43] Deste Gökay G, Oyar P, Gökçimen G, Durkan R. Static and dynamic stress analysis of different crown materials on a titanium base abutment in an implant-supported single crown: a 3D finite element analysis. BMC Oral Health. 2024;24(1):545. 38730391 10.1186/s12903-024-04328-0 PMC11088090

[44] Quaresma SE, Cury PR, Sendyk WR, Sendyk C. A finite element analysis of two different dental implants: stress distribution in the prosthesis, abutment, implant, and supporting bone. J Oral Implantology. 2008;34(1):1–6. 18390236 10.1563/1548-1336(2008)34[1:AFEAOT]2.0.CO;2

[45] Hansson S. Implant-abutment interface: Biomechanical study of flat top versus conical. Clin Implant Dent Relat Res. 2000;2(1):33–41. 11359273 10.1111/j.1708-8208.2000.tb00104.x

[46] Vinayak PO, Manisha H, Viraj P, Shahnawaz M, Megha S, Aquaviva F. Stress distribution in bone and implants in mandibular 6-Implant-Supported cantilevered fixed prosthesis: A 3D finite element study. Implant Dent. 2015;24(6):680–5. 26165389 10.1097/ID.0000000000000300

[47] Baggi L, Pastore S, Di Girolamo M, Vairo G. Implant-bone load transfer mechanisms in complete-arch prostheses supported by four implants: A three-dimensional finite element approach. J Prosthet Dent. 2013;109(1):9–21. 23328192 10.1016/S0022-3913(13)60004-9

[48] Reilly DT, Burstein AH. The elastic and ultimate properties of compact bone tissue. J Biomech. 1975;8(6):393–6. 1206042 10.1016/0021-9290(75)90075-5

[49] Niinomi M. Mechanical properties of biomedical titanium alloys. Mater Sci Eng. 1998;243(1):231–6.

[50] Salem SH, AlSourori AA, Mostafa MH. Effect of thermocycling on acetal resin vs PEEK surface hardness and flexure strength of Implant-Retained overdenture bars. In vitro study. Bull Natl Res Centre. 2023;47:125.

[51] Benzing UR, Gall H, Weber H. Biomechanical aspects of two different implant-prosthetic concepts for edentulous maxillae. Int J Oral Maxillofac Implants. 1995;10(2):188–98. 7744438

[52] Fazi G, Tellini S, Vangi D, Branchi R. Three-dimensional finite element analysis of different implant configurations for a mandibular fixed prosthesis. Int J Oral Maxillofac Implants. 2011;26(4):752–9. 21841984

[53] Liao X, Cao R, Zhong J, Pan Sh C. Influence of implant distribution on the Biomechanical behaviors of mandibular implant-retained overdentures: a three-dimensional finite element analysis. BMC Oral Health. 2024;24(1):405. 38555452 10.1186/s12903-024-04146-4 PMC10981806

[54] Yoon D, Pannu D, Hunt M, Londono J. Occlusal considerations for full-arch implant-supported prostheses: A guideline. Dentistry Rev. 2022;2(2):100042.

[55] Welsch G, Boyer R, Collings EW. Material properties handbook: Titanium alloys. Materials Park, Ohio: ASM International; 1994. https://login.stanford.edu/idp/profile/SAML2/POST/SSO?execution=e1s2

[56] Shruti SH, Shrishail KV, Priyanka T. Application of finite element analysis in dentistry. A review: J Int Oral Health. 2021;13(5):415–22.

